# Therapeutical and Pharmaceutical Notes

**Published:** 1901-02

**Authors:** 


### THERAPEUTICAL AND PHARMACEUTICAL NOTES.

Drug Adulteration. The greed of modern commercialism is aptly illustrated in the report submitted by the Committee on Adulteration of the National Wholesale Druggists' Association, and printed in Myers Brother's *Druggist* for November, 1900. It would appear from the way this report reads that all regard for human or animal life has ceased to be a consideration in the conscienceless mind of some of the drug sophisticators, and that every human tie that should bind the individual to society has been ruthlessly severed or disregarded in the pursuit of the "almighty dollar." The plea set up by these pariahs of commerce, when taken to task for their nefarious conduct, is that the retail pharmacists of the country demand such bogus drugs, and that the salesman of the drug jobber is bluntly informed by the latter that it is quantity and not quality they want. While this assertion may be true of a small percentage of pharmacists, it most assuredly does not apply to the majority of the members of the pharmaceutical profession, who are honorable business men and who would scorn to prostitute their profession to so base a purpose. This assertion is born out by the Committee on Adulteration recommending "that we deprecate the sale of misbranded and adulterated foods and drugs; that we constantly keep before us the importance of quality in the products we sell, on many of which life is often dependent; that we reiterate our indorsement of the Brosius Pure Food and Drug Bill."

It should be the especial care of veterinarians when purchasing or prescribing drugs to see to it that they are obtained from reliable jobbers or pharmacists, because it is only by the use of the very best and purest drugs that disease can be successfully combated and professional success be obtained.

The following are some of the most commonly adulterated drugs, according to the report of the Committee on Adulteration :

"Commercial saltpetre is a mixture of 80 per cent. common salt and 20 per cent. saltpetre. Beeswax which contains 50 to 60 per cent. ceresine is sent out marked 'beeswax,' and is so marked without any other designation. Cottonseed oil is openly sold as Malaga olive oil. If the green is wanted a little chlorophyl is added, and the customer is supplied with this mixture under the label of 'Malaga olive oil, green,' or 'green olive oil.' The so-called black antimony does not contain a grain of antimony, but, instead, is Lehigh soft coal, pure and simple, pulverized. Sometimes we find it of a grayish color, denoting an addition of slate or talc, or something else, not antimony. Borax, a drug largely used in veterinary practice, is often basely adulterated. Some samples recently submitted to analysis by the Pacific Coast Borax Company were found to contain as high as 99 per cent. of soda and not a trace of borax. Oil of sassafras is often largely adulterated with heavy oil of camphor, and oil of cedar, as a genuine pharmaceutical product, has almost entirely disappeared from the market, and its place has been taken by a so-called 'oil of cedar,' which is a fabrication pure and simple. Oil of origanum is even in a worse condition than oil of cedar, for with this article sophistication and manipulation have gone so far as to add asphaltum varnish in order to get body and color. Oil of origanum is quoted by brokers at fifteen to eighty-five cents per pound. Jobbers' prices to retailers, according to a number of price-lists issued, are : Commercial, thirty to thirty-five cents per pound ; pure, forty-five to fifty-five cents per pound. By reference to a quotation of a distiller in France we find oil of origanum quoted at fifteen francs per kilo, or, say, $1.35 per pound at the factory ; yet on this side eighty-five to ninety cents is supposed to represent the cost of the pure oil to the jobber and forty-five to fifty-five cents per pound to the retailer."

Now the facts are, as nearly as can be ascertained by the chairman, that very little true oil of origanum and oil of red cedar are imported at all. Thyme is sold as pure oil of origanum at eighty-five to ninety cents per pound.

BURROW'S ANTISEPTIC SOLUTION. The Medical Society of Anvers has adopted (*Ann. Phar. de Louv.*) the following formula for the official preparation in Belgium of this popular antiseptic solution: Dissolve 100 parts of lead acetate in 300 parts of distilled water; also dissolve 66 parts of potassio-aluminium sulphate and 12 parts of sodium sulphate in 500 parts of distilled water. Mix the two solutions and let stand for two days, then filter.

Starch Poultice. Mix two ounces of potato starch with a small quantity of cold water, and afterward add twenty ounces of boiling water. Boil for an instant, and, when cooling, add two drachms of boric acid.

New Solution of Iodine. Dr. Elsberg (*Philadelphia Medical Journal*) proposes the following solution of iodine in preference to the official tincture: Iodine, twenty grammes; alcohol, forty grammes; ether, forty grammes.

The above is a 20 per cent. solution of iodine in equal parts of alcohol and ether. The point made for the use of this solution in preference to the official tincture is that it dries as rapidly as it is applied on the skin, and is of such strength that but one or two coats need be applied. On account of the alcohol in the official tincture the iodine is spread over a larger area of the skin than is necessary, and it is usually impossible to prevent drops of it from running down the part for some distance and staining it. Because of the rapid evaporation of the alcohol and ether mixture made by the formula here quoted it has no tendency to spread.—*Western Druggist.*

Zomotherapy, or Meat-juice Treatment. Two French physicians, Richet and Hericourt, are advocating (*Deutsche med. Woch.*) the treatment of tuberculosis with expressed meat-juices, calling this method zomotherapy. They claim that the results obtained are due not to the nutrient quality of the food itself, but to some specific cellular secretion contained in the meat-juice. In support of their theory they point to the greater resistance to tuberculosis of carnivorous animals as against herbivorous ones. They also give results of experiments conducted by themselves. They inoculated a number of dogs with tuberculosis more than six months ago. One-third were fed with ordinary food, and all died in three or four weeks; another set with cooked meat, with about the same results; while the third group was fed exclusively with raw meat, and have survived to date of report and are in good health.

